# Genomic stewardship in Alzheimer’s disease: a decade of insights from the NIAGADS platform

**DOI:** 10.1038/s44400-026-00065-z

**Published:** 2026-03-09

**Authors:** Amanda Kuzma, Otto Valladares, Emily Greenfest-Allen, Laura Cantwell, Zivadin Katanic, Maureen Kirsch, Heather Nicaretta, Youli Ren, Heather White, Andrew Wilk, Pavel Kuksa, Wan-Ping Lee, Yuk Yee Leung, Gerard D. Schellenberg, Li-San Wang

**Affiliations:** Penn Neurodegeneration Genomics Center, Department of Pathology and Laboratory Medicine, University of Pennsylvania Perelman School of Medicine, Philadelphia, PA USA

**Keywords:** Computational biology and bioinformatics, Diseases, Genetics, Neuroscience

## Abstract

Fourteen years ago, Alzheimer’s disease (AD) genetics entered an era of exponential data growth, but the infrastructure to support and steward that data had yet to catch up. Large-scale genomic discovery demands more than storage; it requires coordination, ethical rigor, and a platform architecture that transforms raw data into shared knowledge. In response, the National Institute on Aging launched the Genetics of Alzheimer’s Disease Data Storage Site (NIAGADS), not simply to house genetic data for AD and AD-related dementias (ADRD), but to enable its responsible reuse. What began in 2012 as a repository has evolved into an integrated system for policy-aligned access, harmonized data production, and broad community engagement. A detailed overview of NIAGADS was recently published as a Perspective in Alzheimer’s & Dementia^1^. In this Commentary, we reflect on key lessons from building and operating NIAGADS at national scale, with the goal of informing the next generation of genomic platforms.

With the launch of the Alzheimer’s Disease Sequencing Project (ADSP) in 2012, NIAGADS assumed a central role in coordinating the storage and sharing of large-scale Alzheimer’s disease genomic data. As the scale and complexity of ADSP datasets grew, NIAGADS’ data-sharing implementation evolved to support secure, policy-aligned access to whole-genome and other high-volume genomic data, leading to the development of the NIAGADS Data Sharing Service (DSS) as a GDS-compliant repository in 2018.

Over time, this evolution occurred alongside the emergence of a broader ecosystem of complementary data repositories supporting clinical, imaging, and other data modalities (e.g., NACC and LONI). In this context, NIAGADS has focused on enabling interoperability and coordinated data reuse, particularly when cohorts contribute genomic data that are sequenced and harmonized through ADSP, while operating alongside other specialized platforms. As of January 2026, NIAGADS hosts 142 datasets spanning more than 238,000 samples and 23 data types, including whole genome and exome sequences, single nucleotide polymorphism (SNP) arrays, and multi-omics data. Its largest contributors include the Alzheimer’s Disease Sequencing Project (ADSP), the Alzheimer’s Disease Genetics Consortium (ADGC), and the Health and Retirement Study (HRS). These programs contribute data across multiple dimensions, including large numbers of participants recruited by many cohorts, high volume genomic datasets, and sustained longitudinal data submission over time. Many datasets are linked to harmonized clinical, biomarker, and imaging data from national repositories such as National Alzheimer’s Coordinating Center (NACC) and Alzheimer’s Disease Neuroimaging Initiative (ADNI), enabling integrative analyses across diverse data modalities. With roughly 5000 validated registered users and supporting more than 200 distinct Data Access Requests (DARs) from research projects worldwide, NIAGADS plays a critical role in enabling large-scale research and global collaboration in AD genetics research.

The story of NIAGADS reflects a series of decisions, adaptations, and challenges that shaped the platform into what it is today. What follows is a reflection on that journey over a decade of building and sustaining a national genomics infrastructure. Each section highlights a core part of the NIAGADS platform, starting with foundations, followed by operational strategies, and concluding with approaches to community engagement and scientific support (Fig. 1).

**Fig. 1:**
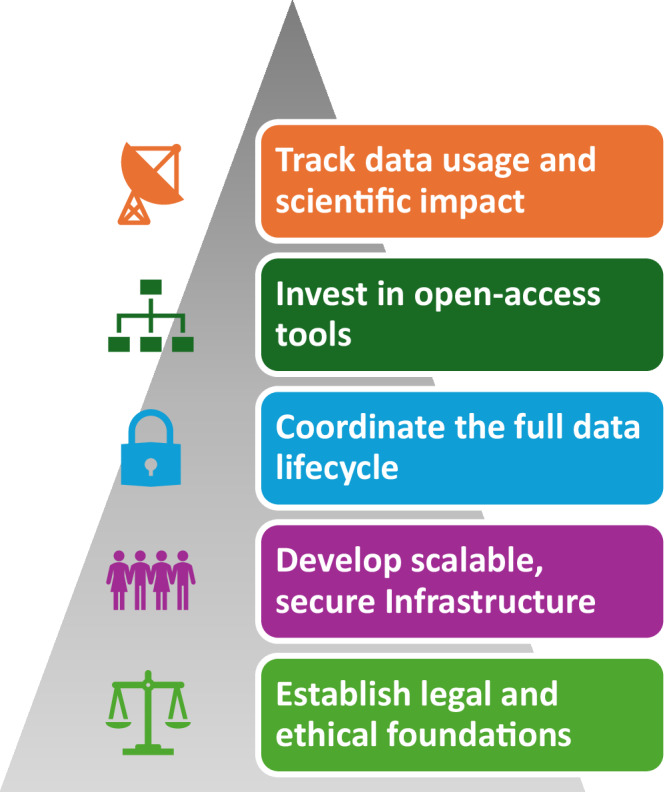
A layered model of the NIAGADS design principles. NIAGADS was designed and operated as a vertically integrated system, with foundational attention to policy alignment and secure access, scalable infrastructure, and coordination across the data lifecycle. These layers enabled open-access tools and broad scientific engagement, culminating in measurable impact through global data reuse and translation. Each layer supports the one above it, reflecting how deliberate platform architecture can enable discovery at scale while maintaining ethical and operational integrity.

## Trust begins with legal and ethical foundations

Trusted genomic data sharing begins with a strong legal and ethical framework. The NIAGADS Data Sharing Service (DSS) platform is anchored in the NIH Genomic Data Sharing (GDS) Policy^2^, which provides legal protection for researchers and ensures participant data are used only within the scope of the original informed consent. Adhering to this policy has helped us protect the trust granted by participants and minimize long-term risk as policy environments evolve. By closely aligning platform design with the legal rationale behind each GDS requirement, NIAGADS implemented a transparent and enforceable access system without compromising on rigor.

DARs are managed and reviewed through the DSS Data Access Request Management (DARM) system. Each DAR must be submitted by a qualified investigator—typically at the rank of assistant professor or higher—and countersigned by the institutional signing official. DARM interfaces with the NIH eRA Commons system to validate both the investigator and signing official, ensuring they have active, institutionally registered accounts. All DARs are reviewed by the NIAGADS ADRD Data Access Committee (NADAC), consisting of NIH senior staff to ensure separation of decision making from the NIAGADS operational team to avoid conflicts of interest. Each dataset includes an Institutional Certification (IC) completed by the submitting principal investigators and their institution’s institutional review board that governs the study. The IC captures research use limitations based on the language of the original informed consent. Together, these steps ensure a clear legal basis for access control and long-term stewardship.

## Design for scalable, secure infrastructure

A robust infrastructure enables long-term stewardship of large and sensitive datasets. NIAGADS currently manages over 10 petabytes of data, including raw submissions from contributing studies, harmonized outputs, and long-term backups. To meet the demands of global data sharing and consortium coordination, NIAGADS adopted a cloud computing strategy that ensures the necessary capacity, bandwidth, and security. Commercial platforms such as Amazon Web Services (AWS) provide scalable and reliable infrastructure, allowing us to serve approved researchers worldwide without the constraints of on-premise hardware. Cloud-based systems also future-proof operations by eliminating the need for hardware refresh cycles and enabling rapid integration of emerging technologies.

Security is a critical dimension of genomic data stewardship. NIAGADS DSS is fully compliant with the Federal Information Security Modernization Act (FISMA) at the Moderate risk level, in accordance with requirements defined by the National Institute of Standards and Technology (NIST)^3^. By leveraging AWS’s FedRAMP-certified environment^4^, NIAGADS avoids the need to manage physical infrastructure such as data center security and hardware lifecycle, significantly reducing operational overhead. The FISMA framework also defines internal security protocols and provides assurance to funders, data contributors, and participants.

With both FISMA and GDS compliance, NIAGADS is currently among the 20 NIH-designated repositories recognized for implementing Security Best Practices for Controlled-Access Data^5^. This distinction reflects both the technical rigor of the platform and an ongoing commitment to secure, policy-compliant genomic data sharing.

## Coordinating the full data lifecycle

Coordination among partners over the full data cycle enables interoperability and coherence in data sharing. Large projects like ADSP involve contributions from multiple institutions, study cohorts, and repositories—each operating under distinct protocols and governance structures. In many consortia, responsibilities are distributed across separate teams. Without deliberate coordination, this complexity can lead to inconsistency, delays, and loss of critical context during handoffs.

NIAGADS occupies a unique position: beyond data sharing, it also coordinates cohort registration and data production for ADSP. A unified data flow maintains continuity, reduces ambiguity, and ensure that each component of the pipeline aligns with the broader goals of the consortium. This includes handling cohort registration and the associated policy documentation that authorizes data sharing; tracking sequencing and analysis pipelines; and facilitating the transfer of data to designated working groups, such as the Genome Center for Alzheimer’s Disease (GCAD) for sequence processing and the Phenotype Harmonization Consortium (PHC) for clinical data harmonization. This *vertical integration* enables early definition and enforcement of data standards, including file formats, identifier schemas, and metadata conventions. Clear handoffs and transparent processes help all partners track progress and align timelines. The deep involvement of NIAGADS in study design and data production has strengthened its ability to structure data for reuse and to support researchers in finding the right datasets for their questions.

NIAGADS also operates within a broader ecosystem of national AD research infrastructure and collaborates with partner repositories, including the National Alzheimer’s Coordinating Center (NACC) for clinical data, the National Centralized Repository for Alzheimer’s Disease and Related Dementias (NCRAD) for biospecimens, the AD Knowledge Portal (ADKP) for omics data, and the Laboratory of Neuro Imaging (LONI) for imaging. ADSP sequencing is conducted at two large-scale genome centers: the American Genome Center at the Uniformed Services University of Health Sciences (TAGC/USUHS) and the John P. Hussman Institute for Human Genomics at the University of Miami (HIHG). Some ADSP cohorts and data modalities span multiple repositories, making interoperability a shared responsibility. NIAGADS plays a key role in maintaining these horizontal partnerships to align data standards, ensure consistent participant identifiers, and coordinate data release timelines.

Through this combination of internal coherence and external alignment, NIAGADS serves as both a reliable operational partner and a trusted national hub for Alzheimer’s disease data coordination.

## Investing in open-access tools

Open access broadens participation and accelerates discovery by lowering barriers to data exploration. While many genomic datasets require controlled access, aggregated data such as genome-wide association study (GWAS) summary statistics and functional annotations of genes and variants can often be shared freely without compromising participant privacy.

To meet these needs, NIAGADS provides these resources through an Open Access Portal and four interoperable knowledgebase platforms designed to deliver curated summaries and functional annotations. The NIAGADS Alzheimer’s Disease Genomics Database (GenomicsDB)^6^ hosts harmonized GWAS summary statistics for AD and related traits, combined with genome browser views and functional annotations for over 438 million variants identified by the ADSP. The Functional Genomics Repository (FILER)^7^ offers the most comprehensive collection of 79,249 human functional genomics annotation tracks across more than 20 public data sources, all paired with machine-readable metadata and compatible with other NIAGADS datasets. The Alzheimer’s Disease Variant Portal (ADVP)^8^ curates and harmonizes top genetic association findings from the literature, encompassing more than 80 cohorts and 8 populations. VariXam^9^ allows researchers to inspect quality metrics for all variants called in ADSP, supporting variant-level prioritization for downstream analyses.

Together, these platforms enable hypothesis generation, facilitate data interpretation, and guide more effective use of controlled-access datasets. All tools are interoperable by design, supported by NIAGADS’s vertically integrated infrastructure and aligned with external repositories. A unified API is under development to support programmatic access across platforms. By investing in open-access tools that bridge internal resources and external standards, NIAGADS strengthens its role as a discovery catalyst and broadens engagement with the AD genomics community.

## Tracking data usage and scientific impact

Understanding how data are used and what impact they have provides essential feedback to platform improvement and accountability. NIAGADS maintains a robust internal system for tracking DARs, citations, and user-reported outputs. Approved DARs are required to submit annual renewal requests with updates on research progress and publications. To assess scientific reuse, we analyzed citation records using PubMed to identify papers that acknowledged NIAGADS-supported grants (e.g., ADGC, GCAD, and NIAGADS itself). All entries were manually reviewed to confirm they made use of data stored at NIAGADS.

We identified 422 peer-reviewed articles published between 2012 and April 2025 that used data from NIAGADS-supported projects and cited the relevant grant numbers. About half of these publications were from research groups outside the original consortia, underscoring NIAGADS’s role in democratizing access and enabling new entrants into the AD genetics field. These publications include landmark GWAS articles, rare variant discoveries, ancestry-informed analyses, interactions with APOE, sex-specific genetic risk factors, polygenic risk scores, and QTL studies involving biomarkers and imaging data.

Collectively these 422 publications have been cited 28,156 times by 12,711 unique articles, demonstrating substantial influence on downstream AD research. The impact extends further: those citing articles have themselves been cited 564,652 times, reflecting the wide propagation of findings derived from NIAGADS-hosted data. Analysis of the articles using NIH iCite^10^ reported 2274 (17.9%) have been referenced in clinical documents (e.g. clinical trials, protocols or guidelines), indicating meaningful translational value and relevance for therapeutic development.

This scientific footprint reflects not only the scale of the repository but also the trust placed in its design, curation, and accessibility. NIAGADS continues to develop new strategies to extend this impact through enhanced usage analytics, better support for derivative data sharing, and clearer pathways for tracking translational outcomes.

## Conclusions and future directions

Our journey with NIAGADS began in 2012 in response to urgent needs in AD genetics research. Over the past twelve years, NIAGADS has grown from a data repository into a global hub and catalyst for discovery, serving as an enabling infrastructure for research collaboration, data integration, and scientific innovation. The path was marked by uncertainty, as technologies evolved, policies shifted, and research priorities changed. Through it all, thoughtful data stewardship has provided a consistent set of guiding principles. Many of our implementations drew from successful models such as the NIH database of Genotypes and Phenotypes (dbGaP) and the Centers for Common Disease Genomics (CCDG) program, whose tested solutions helped us anticipate challenges and avoid missteps. The decisions we made around access governance, infrastructure design, data integration, dissemination, and impact tracking were shaped by the complexities of Alzheimer’s research, but they also reflect a broader philosophy that may inform future national-scale data platforms.

In the past decade, scientific research and collaboration have become increasingly global due to advances in technology and the ability for more widespread data sharing. While NIAGADS operates within established U.S. governance and security frameworks, it supports genomic data from globally distributed cohorts, and its datasets are accessed by researchers worldwide. As a result, genetic findings derived from NIAGADS-hosted data advance Alzheimer’s disease research at an international scale.

Looking ahead, the next generation of Alzheimer’s disease data platforms will need to move beyond enabling access toward supporting analysis-ready and interpretation-ready ecosystems. Continued growth in the scale, complexity, and heterogeneity of Alzheimer’s disease datasets has shifted key bottlenecks from data generation to data integration, analysis readiness, and interpretation. Based on this evolution, NIAGADS is prioritizing the expansion of cloud-based analysis capabilities with reproducible workflows; the use of federated and privacy-preserving approaches to support integration with other distributed data sources; and the application of artificial intelligence and other computational methods to strengthen the connection between data dissemination and knowledge synthesis.

At every stage of innovation, protecting the trust placed by research participants and the public remains the foundational priority. At its core, NIAGADS is about honoring those contributions, supporting the scientific community, and building systems that enable discovery while remaining grounded in ethical stewardship and clear governance foundations.

## Data Availability

No datasets were generated or analyzed during the current study.
